# Strain-dependent regulation of hippocampal long-term potentiation by dopamine D1/D5 receptors in mice

**DOI:** 10.3389/fnbeh.2022.1023361

**Published:** 2022-12-05

**Authors:** Hardy Hagena, Martin Stacho, Arthur Laja, Denise Manahan-Vaughan

**Affiliations:** Department of Neurophysiology, Medical Faculty, Ruhr University Bochum, Bochum, Germany

**Keywords:** hippocampus, mouse, synaptic plasticity, dopamine D1/D5 receptor, strain-dependent, CaOlaHsd, C57Bl/6

## Abstract

The magnitude and persistency of long-term potentiation (LTP) in the rodent hippocampus is species-dependent: rats express more robust and more prolonged LTP in response to a broader afferent frequency range than mice. The C57Bl/6 mouse is an extremely popular murine strain used in studies of hippocampal synaptic plasticity and spatial learning. Recently it was reported that it expresses impoverished LTP compared to other murine strains. Given the important role of the dopamine D1/D5 receptor (D1/D5R) in the maintenance of LTP and in memory consolidation, we explored to what extent strain-dependent differences in LTP in mice are determined by differences in D1/D5R-control. In CaOlaHsd mice, robust LTP was induced that lasted for over 24 h and which was significantly greater in magnitude than LTP induced in C57Bl/6 mice. Intracerebral treatment with a D1/D5R-antagonist (SCH23390) prevented both the early and late phase of LTP in CaOlaHsd mice, whereas only late-LTP was impaired in C57Bl/6 mice. Treatment with a D1/D5R-agonist (Chloro-PB) facilitated short-term potentiation (STP) into LTP (> 24 h) in both strains, whereby effects became evident earlier in CaOlaHsd compared to C57Bl/6 mice. Immunohistochemical analysis revealed a significantly higher expression of D1-receptors in the stratum lacunosum moleculare of CaOlaHsd compared to C57Bl/6 mice. These findings highlight differences in D1/D5R- dependent regulation of strain-dependent variations in hippocampal LTP in C57Bl/6 and CaOlaHsd mice, that may be mediated, in part, by differences in the expression of D1R in the hippocampus.

## Introduction

The hippocampus plays an essential role in spatial and episodic memory (Moscovitch et al., 2016; Miry et al., 2020). A primary cellular mechanism through which such information is recorded and stored by this structure is by means of synaptic plasticity (Manahan-Vaughan, 2017, 2018b). In particular, two highly persistent forms of synaptic plasticity, long-term potentiation (LTP) and long-term depression (LTD), have been proposed to comprise the physiological correlates of long-term memory formation (Citri and Malenka, 2008; Takeuchi et al., 2014; Stacho and Manahan-Vaughan, 2022). Strikingly, whereas rats express robust frequency-dependent hippocampal LTP that lasts for days, weeks and months *in vivo* (Abraham, 2003) freely behaving mice, by contrast, show very moderate and short-lived responses to hippocampal afferent stimulation (Buschler et al., 2012). Strain-dependent differences in the magnitude and persistency of hippocampal LTP have been recently reported in mice (Hagena et al., 2022). The question arises as to whether fundamental differences in hippocampal synaptic plasticity properties are evident in these mouse strains that could explain why they express such prominently different LTP profiles.

Persistent forms of LTP and LTD (>24 h) that require *de novo* protein synthesis provide the putative substrate for long term memory (Smolen et al., 2019). One process through which both the persistency and the protein synthesis-dependency of hippocampal plasticity is enabled is through dopaminergic modulation of hippocampal function. In the hippocampal CA1 region, persistent forms of LTP can be blocked by dopamine D1/D5 receptor (D1/D5R) antagonists, both in hippocampal slices and *in vivo* (Swanson-Park et al., 1999; O’Carroll and Morris, 2004). In addition, the facilitation of hippocampal synaptic plasticity by novel spatial experience in rats, is dependent on dopaminergic D1/D5Rs (Lemon and Manahan-Vaughan, 2006, 2011). Furthermore, pharmacological antagonism of D1/D5Rs in rats disrupts long-term but not short-term storage of newly acquired memories, while pharmacological D1/D5R-activation converts short-term into long-term memory (Rossato et al., 2009; Bethus et al., 2010). Others found an effect of D1/D5R manipulations on early LTP in rat brain slices *in vitro* (Otmakhova and Lisman, 1996). In mice, genetic deletion of D1/D5Rs is associated with reduced both early and persistent LTP in hippocampal slices, as well as with impaired spatial learning (Granado et al., 2007). Little is known about the regulation of LTP by D1/D5Rs in freely behaving mice, however. Here, we compared hippocampal LTP in freely behaving CaOlaHsd and C57Bl/6 mice and investigated to what extent LTP in these strains undergoes modulation by D1R. In addition, we explored if strain-dependent differences in the expression of D1R and D5R occur in the hippocampal region from which electrophysiological recordings were made, namely the CA1 region. We report here, that LTP is greater in magnitude and persists for longer in CaOlaHsd compared to C57Bl/6 mice. Furthermore, the modulation of hippocampal LTP by D1/D5R agonism or antagonism differs between strains, as does D1R expression in hippocampal CA1 region. The differences in D1/D5R regulation of LTP, as well as differences in D1R expression in the hippocampus, may thus comprise a molecular substrate for strain-dependent differences in hippocampal LTP in mice.

## Materials and methods

Experiments were conducted using 7–8-week-old male CaOlaHsd and C57Bl/6 mice (Charles River, Germany and Zentrale Versuchstierhaltung der Medizin (ZVM), Ruhr University Bochum) using established methods (Buschler et al., 2012; Manahan-Vaughan, 2018a). Procedures were performed according to the guidelines of the European Communities Council Directive of September 22nd, 2010 (2010/63/EU) for care of laboratory animals and after approval of the local ethics committee (Bezirksamt Arnsberg). The mice were housed in temperature- and humidity-controlled scantainers (Scanbur, DK) with a 12-h light-dark cycle (light-period from 6 a.m. to 6 p.m.). All mice had *ad libitum* access to food and water.

### *In vivo* electrophysiology

**FIGURE 1:**
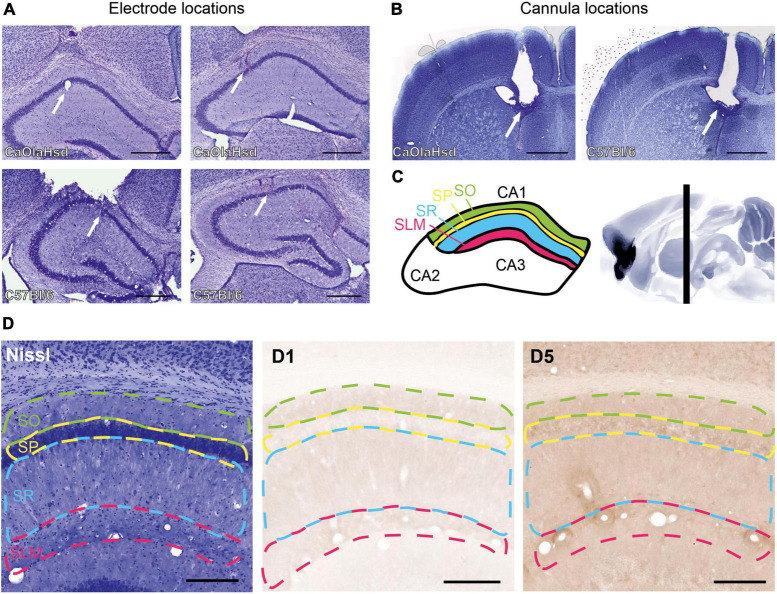
Histological verification of the position of the cannula, recording and stimulation electrodes and illustration of the regions for immunohistochemical analysis. **(A)** Nissl-stained photographs show the position of the monopolar recording electrode in the stratum radiatum in CA1 (white arrow in the left panels) and the bipolar stimulation electrode in the Schaffer-collateral pathway (white arrow in the right panels) in CaOlaHsd (top row) and C57Bl/6 mice (bottom row). Scale bar = 500 μm. **(B)** Nissl-stained photographs show the position of the cannula within the ventricle in CaOlaHsd mice (white arrow in the left panel) and in C57Bl6 mice (white arrow in the right panel). Scale bar = 500 μm. **(C)** Schematic figure of the dorsal hippocampus depicting CA1 delineations used to analyze the laminar distribution of the D1-like receptors. The colored regions correspond to the different layers shown in **(D).** Green: stratum oriens, SO; yellow: Stratum pyramidale, SP; blue: Stratum radiatum, SR and magenta: stratum lacunosum moleculare, SLM). The right panel shows a sagittal cut of the brain. The vertical black bar denotes the position (AP –1.8 mm) used for immunohistochemical analysis. **(D)** Left panel shows a Nissl-stained brain slice that indicates the different layers used for immunohistochemical analysis. The middle and right panels show immunohistochemical stainings of D1 and D5 receptors, respectively, within the same brain slice shown in the left image. Dashed green line: stratum oriens, dashed yellow line: stratum pyramidale, dashed blue line: Stratum radiatum, dashed magenta line: Stratum lacunosum moleculare. Scale bar = 50 μm.

Mice, with a minimum weight of 22 g, were anesthetized with sodium pentobarbital and underwent stereotaxic surgery for chronic implantation of a bipolar stimulation electrode, a monopolar recording electrode and a cannula in the cerebral ventricle at coordinates based on a mouse brain atlas (Watson and Paxinos, 2010), and in line with previous studies by our lab (Hagena et al., 2022). Pre-and postoperative analgesia was implemented using Metacam (Boehringer Ingelheim, Ingelheim am Rhein, Germany) at a dose of 0.01 mL/g bodyweight. The bipolar stimulation electrode was implanted 2.0 mm posterior to bregma (AP) and 2.0 mm lateral to the midline (ML) at a depth of 1.3–1.5 mm from the dura mater, corresponding to the Schaffer collateral (SC) pathway. The recording electrode was implanted in the ipsilateral stratum radiatum of CA1, 1.9 mm AP and 1.4 ML at a depth of 1.1–1.3 mm. The recording electrode was used to monitor evoked potentials at SC-CA1 synapses during implantation (stimulation intensities from 100 to 150 μA). By this means, the final depths of the stimulating and recording electrodes were optimized for each individual animal. A cannula was implanted –0.3 mm AP; 0.9 mm ML and 1.4 mm ventral from the dura mater (Figures 1A–C).

The stimulation and recording electrodes were made of polyurethane-coated stainless steel wire (100 μm diameter; Gündel, BioMedical Instruments, Zöllnitz, Germany). The electrodes were lowered into the brain through a single hole (∼1.6 mm in diameter) that was drilled through the cranial bone. Two additional holes (∼0.7 mm in diameter) were drilled on the contralateral side to insert two anchor screws. Stainless steel wires (A-M Systems Science Products GmbH, Hofheim, Germany), which were attached to the screws, served as reference and grounding electrodes. All five wires were secured to a six-pin socket (Conrad Electronic SE, Hirschau Germany). At the end of the surgery, the socket was fixed onto the skull using dental acrylic (J. Morita Europe GmbH, Dietzenbach, Germany; Haraeus Kulzer GmbH, Dormagen, Germany). After surgery, animals were allowed 10–14 days for recovery. During this time, animals were closely monitored for infections or distress and were handled regularly. Mice were transferred from their housing cages to the recording chambers 24 h before the start of the experiment to ensure familiarization to the environment. The recording chamber measured 80 cm in length x 80 cm in width x 80 cm in height. Animals always had full access to food and water.

Postmortem verifications of electrode (Figure 1A) and cannula localizations (Figure 1B) were conducted. For this, coronal cryosections (30 μm thickness) from the locations of the stimulating and recording electrode, as well as from the ventricle, were prepared and stained with 0.1% cresylviolet. Animals with misplaced electrodes were excluded from analysis.

For recordings of field excitatory postsynaptic potentials (fEPSPs) from the CA1 region, the socket was connected to a flexible cable that was attached via a swivel connector to the recording and stimulation system, allowing for free movement of the animal in the recording chamber (Hagena et al., 2022). The slope of the fEPSP was used to measure changes in synaptic responses that were evoked by stimulation of the SC at a frequency of 0.025 Hz (using single biphasic square waves of 0.2 ms duration per half-wave) generated by a constant current isolation unit (World Precision Instruments, Pfingstweide, Germany). Amplification of the fEPSP signal was performed by a differential AC amplifier (A-M Systems Science Products GmbH, Hofheim, Germany) and digitized through a data acquisition unit (Cambridge Electronic Design, Cambridge, UK). Before each experiment, an input-output (i/o) curve (stimulation intensity of 20, 30, 40, 50, 75, 100, 125, and 150 μA applied at 5-min intervals) was obtained to determine the stimulation intensity used during test-pulse and plasticity -inducing stimulation. This comprised 40% of the intensity that evoked the maximal fEPSP slope during the i/o curve determination. We then evoked potentials from the stratum radiatum of the CA1 region by stimulating the SC with test pulses at 0.025 Hz (five pulses each at 5 min intervals, see section “Electrophysiological data analysis” below) (Buschler et al., 2012). Thirty minutes after beginning of the recordings, vehicle (0.9% NaCl), or a dopamine receptor ligand, was applied. A further 30 min later plasticity-inducing stimulation was applied. The plasticity-inducing stimulation consisted of 4 trains of 50 pulses applied at 100 Hz for high-frequency stimulation (HFS) and 2 trains of 50 pulses at 100 Hz for weak high-frequency stimulation (wHFS) with a 5 min interval between the trains.

### Treatment with dopamine D1/D5 receptor ligands

The D1/5R agonist Chloro-PB (Sigma-Aldrich, Taufkirchen, Germany) was dissolved in 0.9% physiological NaCl solution and injected into the ipsilateral intracerebral ventricle (i.c.v.) via the implanted cannula at a dose of 10.5 μg/μl. The D1/5R antagonist SCH23390 (Tocris Bioscience, Wiesbaden-Nordenstadt, Germany) was dissolved in 0.9% NaCl solution and administered i.c.v at a dose of 7.5 μg/μl. A total amount of 1 μl was injected in 5 min.

### Electrophysiological data analysis

**FIGURE 2:**
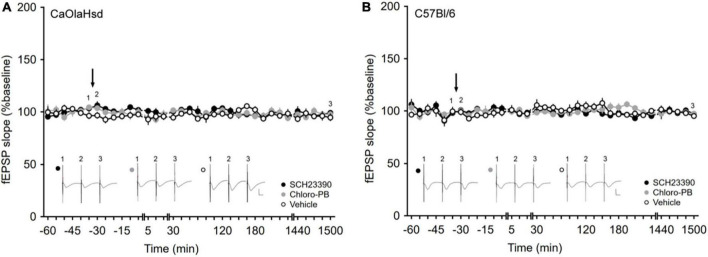
Application of the D1/D5 receptor antagonist and agonist have no influence on basal synaptic plasticity. **(A,B)** Treatment with the D1/D5 receptor antagonist SCH23390 (7.5 μg/μl, solid black circles) or the D1/D5 receptor agonist Chloro-PB (10.5 μg/μl, solid gray circles) has no effect on basal synaptic transmission elicited by test-pulse stimulation compared to vehicle-injection (open circles) in **(A)** CaOlaHsd mice (*p* = 0.24 and *p* = 0.69) and **(B)** C57Bl/6 mice (*p* = 0.20 and *p* = 0.62). Black arrow depicts the time-point of injection. Inset shows analog examples of fEPSPs recorded at the time points indicated by the numbers in the graph. Line breaks indicate change in time-scale. Vertical scale bar corresponds to 2 mV and horizontal scale bar corresponds to 10 ms.

For *in vivo* electrophysiology, each time point that was measured consisted of the average of five consecutive fEPSPs, evoked by test-pulses applied at 0.025 Hz. The first six time points were recorded at 5-min intervals and served as baseline. These six time points were averaged, and all time points throughout the experiment were expressed as the mean percentage ± standard error of the mean (SEM) of this value. Time points were recorded at 5 min intervals until 15 min elapsed after HFS. Afterward the time-points were recorded at 15 min intervals. To determine the longevity of any changes in synaptic plasticity, a further 1 h of recordings (at 15 min intervals) was performed the next day, approximately 24 h after the experiment began. Changes in synaptic transmission were determined by measuring the slope obtained on the first negative deflection of the evoked fEPSP. The stability of synaptic transmission (in the presence of vehicle, or D1/5R ligand) was assessed by conducting an experiment for the same duration of a plasticity experiment, but in the absence of any plasticity-inducing stimulation (Figure 2).

**FIGURE 3:**
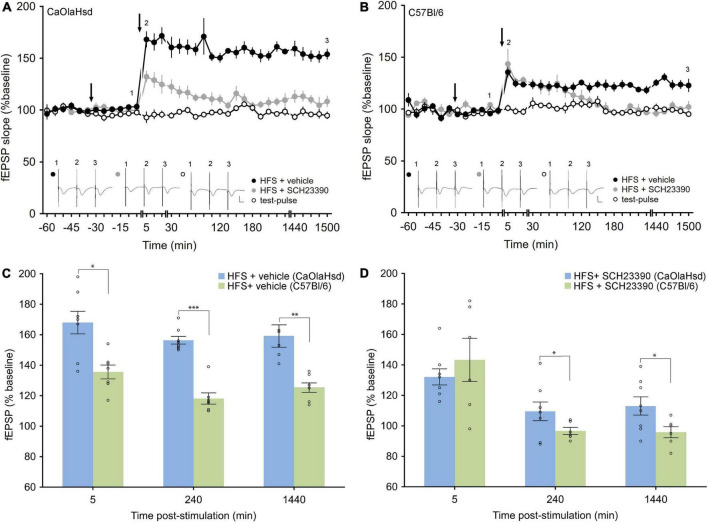
Effect of the D1/5 receptor antagonism on LTP in CaOlaHsd and C57Bl/6 mice. **(A)** High frequency stimulation (HFS) of the SC-CA1 pathway **(second arrow)** results in LTP in CaOlaHsd mice **(solid black circles)** that was significant compared to test-pulse stimulation (*p* < 0.001, **open black circles**). Treatment with the D1/D5 receptor antagonist **(first arrow)**, results in an impairment of the early and late phase of LTP (*p* < 0.001, **solid gray circles**). Inset shows analog traces recorded at the time points indicated by the numbers in the graph. Line breaks indicate change in time-scale. Vertical scale bar corresponds to 2 mV and horizontal scale bar corresponds to 10 ms. **(B)** HFS of the SC-CA1 pathway **(second arrow)** results in a smaller and less persistent LTP in C57Bl/6 mice compared to CaOlaHsd mice **(solid black circles)** that was significantly different compared to test-pulse stimulation (open black circles). Injection of the D1/D5 receptor antagonist **(first arrow)**, resulted in an impairment of the late phase of LTP **(solid gray circles)**. Inset shows analog traces recorded at the time points indicated by the numbers in the graph. Line breaks indicate change in time-scale. Vertical scale bar corresponds to 2 mV and horizontal scale bar corresponds to 10 ms. **(C,D)** Bar charts display the difference in evoked responses between CaOlaHsd (blue) and C57Bl/6 mice (green) at different time-points after stimulation in LTP control experiments **(C)** and after injection of SCH23390 **(D)**. Open circles represent single data-points. Significant values are denoted by asterisks: *p < 0.05, ^**^p < 0.01 and ^***^p < 0.001.

**FIGURE 4:**
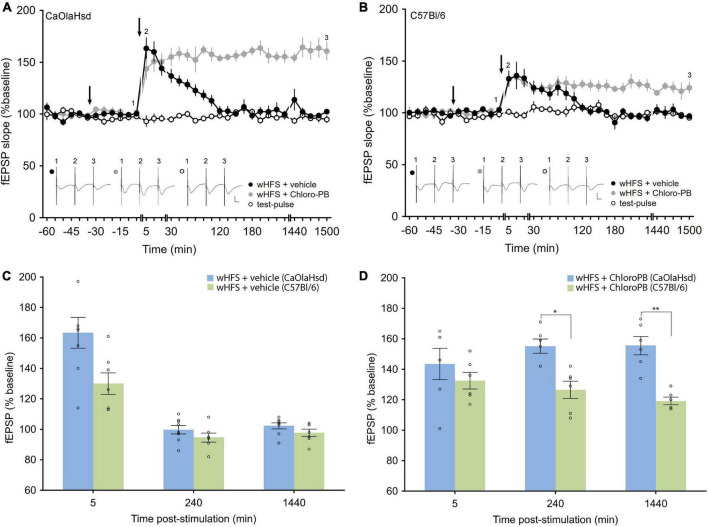
Effect of the D1/5 receptor agonist Chloro-PB on LTP in CaOlaHsd and C57Bl/6 mice. **(A)** Weak high frequency stimulation **(wHFS, second arrow)** results in STP in CaOlaHsd mice (solid black circles) that was significant compared to baseline (test-pulse only) responses for 2 h after wHFS (open black circles). Treatment with the D1/D5 receptor agonist Chloro-PB 30 (first arrow), results in a facilitation of LTP (solid gray circles). Inset shows analog traces recorded at the time points indicated by the numbers in the graph. Line breaks indicate change in time-scale. Vertical scale bar corresponds to 2 mV and horizontal scale bar corresponds to 10 ms. **(B)** Stimulation of the SC-CA1 pathway with wHFS **(second arrow)** resulted in an initially smaller STP in C57Bl/6 mice **(solid black circles)** (compared to CaOlaHsd mice) that was nonetheless significantly different compared to test-pulse stimulation **(open black circles)**. Treatment with the D1/D5 receptor agonist Chloro-PB **(first arrow)**, resulted in a facilitation of LTP **(solid gray circles)**. Inset shows analog traces recorded at the time points indicated by the numbers in the graph. Line breaks indicate change in time-scale. Vertical scale bar corresponds to 2 mV and horizontal scale bar corresponds to 10 ms. **(C,D)** Bar charts display the difference in evoked responses between CaOlaHsd **(blue)** and C57Bl/6 mice **(green)** at different time-points after stimulation in STP control experiments **(C)** and after injection of Chloro-PB **(D)**. Open circles represent single data-points. Significant values are denoted by asterisks: *p < 0.05, **p < 0.01.

All statistical tests were performed using STATISTICA 13 (Statsoft, Tulsa, Oklahoma, USA). Between-group effects were assessed by means of repeated measures analysis of variance (ANOVA). ANOVA was assessed for all time-points after HFS or wHFS (beginning at time-point 5 min and ending with the final timepoint of the experiment). A *post hoc* Fisher‘s test was used to discriminate significant effects at specific time-points after HFS or wHFS. This was done to determine when a significant difference in plasticity responses became no longer significant. In the case, where differences between the early phase of LTP (time-point 1), late-LTP (time-point 2) and late late-LTP (time-point 3) were assessed (see Figures 3C,D, 4C,D), a Student’s *t*-test was employed for the comparison of responses in CaOlaHsd and C57Bl/6 mice. All data are reported as mean ± standard error of mean The significance levels were set at *p < 0.05, ^**^p < 0.01, and ^***^p < 0.001.

### Immunohistochemistry

To evaluate whether the contribution of D1R and D5R to strain-dependent differences is determined by differences in receptor expression, immunostainings were conducted using an avidin-biotin complex (ABC) method as described previously (Heras et al., 1995; Dubovyk and Manahan-Vaughan, 2018; Feldmann et al., 2019; Beckmann et al., 2020). To verify the specificity of antibody binding, tissue incubations with separate primary and secondary antibodies served as negative controls (Feldmann et al., 2019). The immunostainings were done on sections containing the dorsal CA1 at AP coordinates of –1.7 to –2.18 mm (Watson and Paxinos, 2010; Figures 1C,D).

Tris-buffered saline (TBS) and phosphate-buffered saline (PBS) were used as buffer solutions for D1R and D5R immunostainings, respectively. Bovine serum albumin (BSA, Sigma-Aldrich, St. Louis, USA) and normal goat serum (NGS, Vector Laboratories, Newark, CA, USA) were used as blocking serums for D1R and D5R immunostainings, respectively. After rinsing thrice in buffer, sections were placed in 0.3% H_2_0_2_ for 20 min to quench endogenous peroxidase activity, so as to reduce background staining. Then, they were rinsed a further three times before pre-incubation in a solution containing 20% avidin (avidin-biotin blocking kit, Vector Laboratories, Newark, CA, USA), 10% blocking serum, and 0.2% Triton X-100 (Tx) for 90 min to reduce non-specific binding. Subsequently, the sections were incubated overnight at room temperature with the primary antibody solution, which contained 20% biotin (avidin-biotin blocking kit, Vector Laboratories, Newark, CA, USA), 1% blocking serum, 0.2% Tx, and the primary antibody with these concentrations: D1R (1:100, rabbit polyclonal, ADR-005, Alomone Labs, Jerusalem, Israel), D5R (1:500, rabbit polyclonal, AB1765P, Sigma-Aldrich, Darmstadt, Germany). The sections were then rinsed thrice in buffer before being incubated for 90 min in the secondary solution, which contained 1% blocking serum, 0.1% Tx (for D1R)/0.2% Tx (for D5R), and biotinylated goat anti-rabbit (B-GAR, rabbit, 1:500, BA-1000, Vector Laboratories, Newark, CA, USA). For D5R, the ABC-Elite detection system was applied 1:1,000 for 90 min. For D1R, the ABC-Elite detection system was applied for 30 min, and after quick rinsing in buffer, the detection system was enhanced with biotinylated tyramide for 20 min before applying the ABC-Elite detection system for an added 30 min. The reaction was visualized by incubating the sections in 0.05% 3, 3′-Diaminobenzidine-solution (DAB, Sigma-Aldrich, St. Louis, USA) in PBS and 0.01% H_2_0_2_ for 10 min for D1R and 5 min for D5R.

To analyze the D1R and D5R receptor distribution of the dorsal CA1, optical density was measured using the software ImageJ, Fiji (Schindelin et al., 2012). Sections were imaged using an Axioscan Z1 (Zeiss, Jena, Germany) equipped with an HV-F202SCL (Hitachi Kokusai Electric Inc., Tokyo, Japan) camera using an EC PLAN-NEOFLUAR 20 × 0.50 M27 objective (Zeiss, Jena, Germany). The analysis explained below is similar to the approach reported in Dubovyk and Manahan-Vaughan (2018).

The optical tract, or reticular nucleus in the prethalamus, were used to correct for background staining intensity differences between sections. The corrections were done by taking a measurement of the correction area and subtracting it from every measurement from all sub-regions of the CA1 for each individual slice. The sub-regions of the CA1 were found by using a neighboring Nissl-stained section and delineating the immunostained section accordingly. Measurements were taken in ImageJ from images with the red, green and blue (RGB), color type enabled. The “Color Deconvolution” plugin was used on the images with the vector “[H DAB]” used to isolate the DAB color signal. The images were then converted to grayscale and inverted to obtain luminance information ranging from 0 (black) to 255 (white). Finally, the R software package “blotIt” was used to scale data from independent stainings using a generalized residual sum of squares algorithm to account for variance of staining intensity across batches^1^ (Kreutz et al., 2007; Heyde et al., 2014; Dubovyk and Manahan-Vaughan, 2018; Kemmer et al., 2022). The blotIt-scaling procedure was done using its default options, and this process served to normalize the data so that the mean was 1 for all conditions. Statistical analyses were performed using R as well as the open-source statistical software JASP (JASP Team, Version 0.16.1). All data are reported as mean ± standard deviation. One-way ANOVAs were run to analyze the immunohistochemical results of D1R and D5R separately.

## Results

### Basal synaptic transmission is stable and unaffected by dopamine D1/D5 receptor-ligand treatment in both mouse strains

Basal synaptic transmission, evoked by test-pulse stimulation, remained stable in both CaOlaHsd (*n* = 8, Figure 2A) and C57Bl/6 mice (*n* = 7, Figure 2B) over a 25 h monitoring period. We then tested the effects of intracerebral treatment with a D1/D5R-antagonist, SCH23390, 7.5 μg) and a D1/D5R-agonist (Chloro-PB, 10.5 μg), on basal synaptic transmission. Here, neither D1/D5R-ligand affected basal synaptic transmission that remained stable over the entire monitoring period after ligand administration in both CaOlaHsd (*n* = 7, Figure 2A) and C57Bl/6 mice (*n* = 6 for SCH23390-treated and *n* = 7 for Chloro-PB-treated animals, Figure 2B). ANOVA for SCH23390 compared to baseline in CaOlaHsd and C57Bl6, revealed the following outcome: for CaOlaHsd: *F*_(1_, _13)_ = 1.52, *p* = 0.24; interaction effect: ANOVA: *F*_(22_, _286)_ = 1.15, *p* = 0.30. For C57Bl/6: *F*_(1_, _11)_ = 1.90, *p* = 0.20; interaction effect: ANOVA: *F*_(22_, _242)_ = 0.91, *p* = 0.59). ANOVA for Chloro-PB compared to baseline in CaOlaHsd and C57Bl6, respectively: *F*_(1_, _13)_ = 0.17, *p* = 0.69; interaction effect: ANOVA: *F*_(22_, _286)_ = 0.91, *p* = 0.58; *F*_(1_, _12)_ = 0.25, *p* = 0.62; interaction effect: ANOVA: *F*_(22_, _264)_ = 1.77, *p* < 0.05.

### Hippocampal long-term potentiation in freely behaving mice is weaker in C57Bl/6 compared to CaOlaHsd mouse strains

We then tested hippocampal LTP in both mouse strains. We stimulated the Schaffer collaterals with high frequency stimulation (HFS) at 4 × 50 pulses at 100 Hz, as we had previously established that this gives rise to LTP that last for more than 24 h in freely behaving C57Bl/6 mice (Ballesteros et al., 2016). Here, HFS resulted in LTP in vehicle-treated CaOlaHsd mice (*n* = 8) that lasted for over 24 h and that was significantly larger compared to C57Bl/6 (*n* = 7) mice whose LTP also lasted for > 24 h [ANOVA: *F*_(1_, _13)_ = 54.39, *p* < 0.001; interaction effect: *F*_(22_, _286)_ = 0.97, *p* = 0.51; Figures 3A,B]. The Fisher’s LSD *post hoc* analysis confirmed significantly higher responses in CaOlaHsd mice for every time-point after HFS until the end of the experiment. A comparison of evoked responses between CaOlaHsd and C57Bl/6 mice after vehicle treatment (control groups) revealed significant differences 5 min [*t*(13) = 3.23, *p* < 0.05], 3 h [*t*(13) = 7.95, *p* < 0.001] and 24 h [*t*(13) = 5.03, *p* < 0.01] after HFS (Figure 3C).

### Hippocampal long-term potentiation is modulated to different extents by dopamine D1/D5 receptor -ligands in C57Bl/6 compared to CaOlaHsd mouse strains

Treatment with the D1/D5R-antagonist, SCH23390 (7.5 μg), significantly prevented LTP in both mouse strains (Figures 3A,B), but whereas both early and late phases were impaired in CaOlaHsd mice [ANOVA: *F*_(1_, _14)_ = 73.23, *p* < 0.001; interaction effect: ANOVA: *F*_(22_, _308)_ = 0.60, *p* = 0.93; *n* = 8 in both groups], only the later phases of LTP were affected in C57Bl/6 mice [ANOVA: *F*_(1_, _11)_ = 60.86, *p* < 0.001; interaction effect: ANOVA: *F*_(15_, _165)_ = 1.84, *p* < 0.05; *n* = 6 in the SCH23390 group and *n* = 7 in the control group]. The Fisher’s LSD *post hoc* test revealed that the impairment effect on LTP became significant 2 h after HFS (Figures 3A,B,D). Evoked responses between CaOlaHsd and C57Bl/6 mice after treatment with the D1/D5R-antagonist SCH23390, revealed significant differences 3 h [*t*(12) = 3.08, *p* < 0.05] and 24 h [*t*(12) = 2.64, *p* < 0.05] but not 5 min [*t*(12) = –0.8, *p* = 0.46] after HFS (Figure 3D).

In rats, intracerebral treatment with a D1/D5R-agonist facilitates short-term potentiation (STP) into long-term potentiating *in vivo* (Swanson-Park et al., 1999; O’Carroll and Morris, 2004; Lemon and Manahan-Vaughan, 2006). We examined whether similar effects are evident in mice. First we tested the effects of a weaker submaximal HFS (wHFS, 2 trains of 50 pulses at 100 Hz, Ballesteros et al., 2016) in the presence of vehicle. Here, STP was elicited in both strains with equal magnitude in CaOlaHsd (*n* = 8) and C57Bl/6 mice [*n* = 7, ANOVA: *F*_(1_, _13)_ = 2.60, *p* = 0.13; interaction effect: ANOVA: *F*_(22_, _286)_ = 1.78, *p* < 0.05; Figures 4A–C). A comparison between CaOlaHsd and C57Bl/6 mice after vehicle treatment revealed no significant differences in the evoked responses 5 min [*t*(13) = 2.30, *p* = 0.06], 3 h [*t*(13) = 1.80, *p* = 0.12] and 24 h [*t*(13) = 1.23, *p* = 0.27] after HFS (Figure 4C).

Treatment with a D1/D5R-agonist (Chloro-PB, 10.5 μg), significantly facilitated STP into LTP in both strains [ANOVA for CaOlaHsd: *F*_(1_, _12)_ = 101.69, *p* < 0.001; interaction effect: ANOVA: *F*_(22_, _264)_ = 7.96, *p* < 0.001; and ANOVA for C57Bl/6: *F*_(1_, _11)_ = 13.41, *p* < 0.001; interaction effect: ANOVA: *F*_(22_, _242)_ = 4.47, *p* < 0.001], whereby effects were more pronounced in CaOlaHsd (*n* = 6) compared to C57Bl/6 mice (*n* = 6) [ANOVA: *F*_(1_, _10)_ = 40.10, *p* < 0.001; interaction effect: ANOVA: *F*_(22_, _220)_ = 0.97, *p* = 0.51]. The *post hoc* analysis revealed significantly higher responses in CaOlaHsd compared to C57Bl/6 mice, 15 min after wHFS. Furthemore, *post hoc* analysis determined that the effect of agonist-treatment became significant 30 min after wHFS in CaOlaHsd mice, whereas significant effects were first observable 2 h after wHFS in C57Bl/6 mice (Figures 4A,B,D). A comparison of evoked responses between CaOlaHsd and C57Bl/6 mice after treatment with the D1/D5R-agonist Chloro-PB, revealed significant differences 3 h [*t*(10) = 2.79, *p* < 0.05] and 24 h [*t*(10) = 5.75, *p* < 0.01] but not 5 min [*t*(10) = 1.51, *p* = 0.19] after HFS (Figure 4D).

### D5R expression is equivalent and D1R expression differs in C57Bl/6 and CaOlaHsd mouse strains

We then asked the question whether differences in D1/D5R expression in the CA1 region could explain the results we had obtained in the abovementioned synaptic plasticity experiments. For this immunohistochemical analysis of the expression of D1R and D5R in CA1 was performed.

**FIGURE 5:**
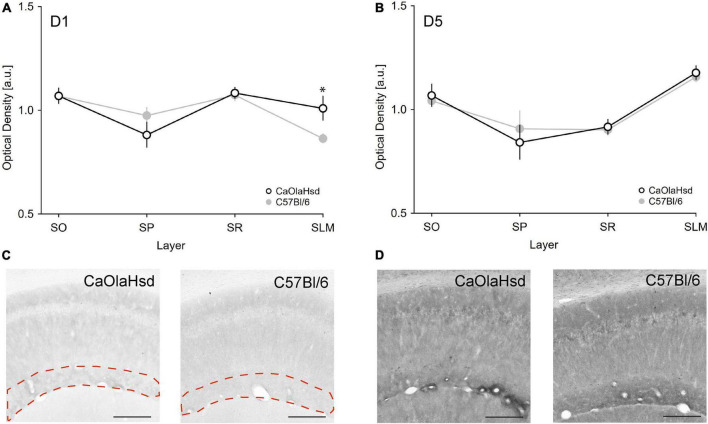
Expression of D1 and D5 receptors are different in CaOlaHsd and C57Bl/6 mice. **(A,B)** The graphs depict the optical density of D1R **(A)** and D5R **(B)** expression in different layers of CA1 in CaOlaHsd **(open black circles)** and C57Bl/6 **(closed gray circles)** mice. D1 receptor expression is significantly higher in CaOlaHsd mice compared to C57Bl/6 mice in SLM **(A)**. The CA1 sublayers assessed show no differences with regard to D5 receptor expression in CaOlaHsd mice compared to C57Bl/6 mice **(B)**. SLM: stratum lacunosum moleculare, SO: stratum oriens, SP: stratum pyramidale, SR: stratum radiatum. **(C,D)** Immunostainings of the different layers in the CA1 region of CaOlaHsd and C57Bl/6 mice. The dotted magenta lines in **(C)** outline the stratum lacunosum moleculare, where D1R **(C)** showed increased expression in CaOlaHsd mice. No differences in expression of D5R were detected between strains **(D)**. Scale bar = 200 μm. Significant values are denoted by an asterisk. *p < 0.05.

Analysis of D5R expression revealed no significant differences between C57Bl/6 and CaOlaHsd mouse strains for all tested layers (Table 1 and Figure 5). A different picture emerged with respect to D1R. Here, a comparison of the expression of these receptors revealed a significantly higher expression of D1R in the stratum lacunosum moleculare in CaOlaHsd compared to C57Bl6 mice [ANOVA: *F*(1, 10) = 6.25, *p* < 0.05, *n* = 6 in each group) (Figure 5 and Table 1). A detailed statistical analysis of D1R and D5R expression within the different layers in CA1 is shown in Table 1.

**TABLE 1:** Statistical comparison of D1 and D5 receptor expression within the layers of CA1 between CaOlaHsd (*n* = 6) and C57Bl/6 mice (*n* = 6).

	Layer	C57Bl/6	CaOlaHsd	ANOVA	η^2^
		Optical density [a.u.] (SD)	Optical density [a.u.] (SD)		
D1	SO	1.069 (0.089)	1.069 (0.093)	*F*_(1_, _10)_ = 4.487e-5, *p* = 0.995	0.000
	SP	0.975 (0.095)	0.881 (0.150)	*F*_(1_, _10)_ = 2.582, *p* = 0.116	0.205
	SR	1.073 (0.072)	1.083 (0.067)	*F*_(1_, _10)_ = 0.028, *p* = 0.869	0.003
	SLM	0.863 (0.057)	1.009 (0.142)	*F*_(1_, _10)_ = 6.254, *p* = 0.017*	0.385
D5	SO	1.042 (0.058)	1.068 (0.133)	*F*_(1_, _10)_ = 0.119, *p* = 0.732	0.012
	SP	0.907 (0.211)	0.841 (0.204)	*F*_(1_, _10)_ = 0.810, *p* = 0.373	0.075
	SR	0.902 (0.067)	0.916 (0.089)	*F*_(1_, _10)_ = 0.035, *p* = 0.853	0.004
	SLM	1.157 (0.063)	1.177 (0.082)	*F*_(1_, _10)_ = 0.070, *p* = 0.792	0.007

Main effects were analyzed with strain as the “simple effect” factor and “layer” as the moderating factor. Optical Density [a. u.] from all sublayers presented as mean values. Significant values are denoted by an asterisk.

*p < 0.05. a.u., arbitrary units; SLM, stratum lacunosum moleculare; SO, stratum oriens; SP, stratum pyramidale; SR, stratum radiatum.

## Discussion

In this study, we explored to what extent strain-dependent differences in synaptic plasticity that occur in different mouse strains can be explained by differences in D1/D5R neuromodulation. We report that strain-dependent differences in the magnitude of both LTP and STP occur in the hippocampus of freely behaving mice, whereby LTP in CaOlaHsd mice is more robust than LTP in C57Bl/6 mice. We also observed that the modulation of hippocampal synaptic potentiation by dopaminergic D1/D5Rs in freely behaving mice varies depending on the mouse strain scrutinized: Whereas both early and late phases of LTP and STP are regulated by dopaminergic D1/D5Rs in CaOlaHsd mice, only the late phase of LTP is regulated by D1/D5R in C57Bl/6 mice. To clarify whether these differences might be explained by differences in the relative expression of D1R or D5R in the hippocampus, we used an immunohistochemical approach to study receptor expression in the hippocampal CA1 region, where LTP had been recorded. We observed that whereas D5R expression is equivalent in both strains, D1R expression is higher in the stratum lacunosum moleculare of CaOlaHsd compared to C57Bl/6 mice.

Recent studies showed that profound differences in hippocampal synaptic plasticity, receptor expression and learning exist between different mouse strains, whereby C57Bl/6 mice show marked deficits in both hippocampal synaptic plasticity and hippocampus-dependent learning compared to other strains (Feldmann et al., 2019; Beckmann et al., 2020). In these studies it was shown that LTP is markedly impaired in 4-month-old C57Bl/6 compared to CaOlaHsd mice. This difference is also associated with strain-dependent differences in hippocampus-dependent learning that involve a reasonably high cognitive demand such as remembering the spatial position of objects (item-place test). Here, C57Bl/6 mice showed impaired performance in recognizing the novel spatial configuration compared to CaOlaHsd mice (Beckmann et al., 2020). The C57Bl/6 mouse strain develops hearing loss in early adulthood, and progressively loses the ability to hear auditory frequencies, beginning with higher frequencies at 2 months and progressing steadily downwards in frequency bands, ranging from ultrasonic through sonic ranges, until reaching complete deafness at the age of 24 months (Mikaelian, 1979; Henry and Chole, 1980; Shnerson and Pujol, 1981; Shnerson et al., 1983; Park et al., 2010). This loss in sensory perception may be one reason for the impairment in synaptic plasticity (Feldmann et al., 2019; Beckmann et al., 2020).

D1/D5 receptors play a profound role in the induction and maintenance of LTP (Frey et al., 1990; Frey and Morris, 1998; Sajikumar and Frey, 2004) which constitutes a key cellular mechanism for hippocampus-dependent long-term memory formation (Kemp and Manahan-Vaughan, 2004; Pastalkova et al., 2006; Hagena and Manahan-Vaughan, 2011; Nabavi et al., 2014). We explored whether the differences in LTP in C57Bl/6 and CaOlaHsd mice might result from species-dependent differences in modulation of LTP through D1/D5R. We found that although the absolute modulation of LTP by D1/D5R is similar between strains, the relative modulation is different: Thus, LTP is curtailed by D1/D5R-antagonism and STP is prolonged into LTP by D1/D5R-agonism in both strains, but early (and late) LTP is impaired by D1/D5R antagonism in the CaOlaHsd strain, whereas only late LTP is impaired by the antagonist in the C57Bl/6 strain. These findings suggest that differences in the modulation by D1/D5R may be present in these two mouse strains.

Our observation that synaptic potentiation is prolonged by D1/D5R-agonism and that LTP is impaired by D1/D5R-antagonism are consistent with previous observations in rats showing that D1/D5Rs facilitate persistent LTP in CA1 (Swanson-Park et al., 1999; O’Carroll and Morris, 2004; Lemon and Manahan-Vaughan, 2006). Similar results were also observed for LTD in the CA1 region of rats where it was reported that LTD that is facilitated by the exploration of a novel spatial object configuration is prevented by D1/D5R-antagonism (Lemon and Manahan-Vaughan, 2006). Moreover, D1/D5R are especially involved in long-term memory (Rossato et al., 2009; Bethus et al., 2010) and spatial recognition learning is impaired by D1/D5R-antagonism (Lemon and Manahan-Vaughan, 2006, 2011) in rats.

But if D1/D5R decisively contribute to the observed strain-dependent differences that are evident in the late phase of LTP, it may be the case that expression of these receptors differs between mouse strains. We implemented immunohistochemical analysis of the hippocampal subregion from which LTP recordings were made. We found no differences in receptor expression with regard to D5R. By contrast, a significantly greater density of D1R was found in the stratum lacunosum moleculare (SLM) in CaOlaHsd compared to C57Bl/6 mice. No expression differences of D1R were found in any other subcompartment of the CA1 region, however. The SLM receives a direct afferent input from layer III of the entorhinal cortex (EC) via the perforant path (pp) (Steward and Scoville, 1976; Witter et al., 1988; Ishizuka et al., 1990) that terminates on the *distal* part of apical dendrites of CA1 (Kajiwara et al., 2008). We have reported in the past that tetanic stimulation of this direct pathway from the EC evokes LTP in CA1 in rats (Aksoy-Aksel and Manahan-Vaughan, 2013). However, the stimulating electrodes were placed in the stratum radiatum in the present study to stimulate the SC pathway. The question is therefore how a higher expression of D1R in the SLM of CA1 in CaOlaHsd mice may contribute to the differences we detected in synaptic plasticity at SC in stratum radiatum.

Dopaminergic activity inhibits responses in CA1 evoked by the stimulation of the temporoammonic (TA) input, but not SC, *in vitro* (Otmakhova and Lisman, 1999, 2000). Lisman and Otmakhova suggested a model in which an increase in DA release would favor the SC input and weaken afferent input via the TA (Lisman and Otmakhova, 2001). Correspondingly, application of an D1/D5R agonist diminishes the TA input to CA1 (Otmakhova and Lisman, 1999, 2000), resulting in a shift toward the SC input and a facilitation of LTP at this input (Frey et al., 1993; Otmakhova and Lisman, 1996; Swanson-Park et al., 1999). Both mouse strains did indeed exhibit improved LTP following agonist activation of D1R in the present study. Due to the higher expression of D1R in the SLM in CaOlaHsd mice, the effect of D1R receptor activation might be stronger in this strain compared to C57Bl/6 mice under endogenous conditions, however, and might therefore serve to explain the fact that freely behaving CaOlaHsd mice express LTP of a much greater magnitude and stability compared to C57Bl/6 mice. Furthermore, the higher expression of D1R may account for the effects of the agonist on the early phase of LTP observed in CaOlaHsd compared to C57Bl/6 mice, where effects are limited to the late phases of LTP.

Extrapolating from the Lisman model, treatment with a D1/D5R antagonist might be expected to *impair* the inhibitory effect of dopamine on the TA input within the SLM and therefore diminish the shift toward information flow through the SC input, thereby weakening LTP at SC-CA1 synapses. Following this line of thinking, the effect of D1/D5R antagonism would arguably be greater in CaOlaHsd mice, given the fact that they express more D1R in the SLM than C57Bl/6 mice. This interpretation aligns with our findings that D1R antagonism elicits a more potent impairment of LTP in the CaOlaHsd strain.

It is noteworthy, however, that the initial levels of LTP in CaOlaHsd mice during D1/D5R antagonism are similar in magnitude to the early LTP in C57Bl/6 that remains unaffected by D1/D5R antagonism (Figures 3A,B,D). This suggests that a shift in the CA1 input toward the SC is negligible in C57Bl/6 mice. However, other factors may play a role. For example, the early effect on LTP of D1/D5R-antagonism might be explained by the interaction of D1/D5R with N-methyl-D-aspartate receptors (NMDAR). Previously it was shown in a C57Bl/6 substrain that the HFS protocol used in the present study to induce LTP, recruits the contribution of GluN2B subunit-containing NMDAR into LTP (> 24 h) (Ballesteros et al., 2016). The activation of D1/D5R in the hippocampus increases the phosphorylation of the GluN2B subunit at the serine residue ser1303 (Sarantis et al., 2009). This residue has been implicated in a protein kinase C-mediated increase in NMDAR currents (Liao et al., 2001). As there is a higher expression of GluN2B subunit in the CA1 region in C57Bl/6 in comparison to CaOlaHsd mice (Beckmann et al., 2020) it is tempting to speculate that this difference may explain why early LTP was resistant to D1/D5R -antagonism in C57Bl/6 mice. Thus, even when D1/D5Rs are antagonized, GluN2B-containing receptors will be activated by HFS, thereby compensating for the lack of dopaminergic receptor activation in C57Bl/6, compared to CaOlaHsd mice. Such a compensation of early but not late phases of LTP in C57Bl/6 mice, suggests that D1/D5R activation is crucial for long-lasting LTP but is less critical for early phases of LTP in this strain. Congruently, in rats, D1/D5Rs are necessary for the persistency but not induction of LTP (Swanson-Park et al., 1999; O’Carroll and Morris, 2004; Lemon and Manahan-Vaughan, 2006).

Other potential routes of strain-dependent modulation by D1/D5Rs of hippocampal LTP are also possible. For instance, D1/D5R-activation can increase α-amino-3-hydroxy-5-methyl-4-isoxazolepropionic acid receptor (AMPAR) conductivity through interactions with GluR1 subunits (Lee et al., 2000; Smith et al., 2005; Derkach et al., 2007; Sarantis et al., 2009), or decrease NMDAR-currents by interacting with GluN2A subunits (Lee et al., 2000; Ladepeche et al., 2013). Furthermore, the effects of D1/D5R-activation might be greater depending on concurrent NMDAR activation (Sarantis et al., 2009). Thus, D1/D5R effects on synaptic plasticity might not only derive from differences in the relative expression in hippocampal synaptic subcompartments, but may also derive from differences in DA modulation of the SLM and SC inputs to the CA1 region, as well as strain-dependent differences in the modulation by D1/D5R of, for example, AMPAR and NMDA receptor efficacy.

Although the findings of our study highlight the contribution of D1/D5R to these processes, strain-dependent differences in LTP between the mouse strains assessed here are not likely to *solely* derive from differences in D1/D5R expression and neuromodulation. In contrast to the CaOlaHsd mouse strain that shows no sensory deficits and consistently stable hippocampal LTP throughout its lifetime (Brooks et al., 2004, 2005; Hagena et al., 2022), the C57Bl/6 strain experiences early loss of hearing that commences at 4 postnatal weeks with the loss of its ability to perceive high ultrasonic frequencies, and progresses through the loss of perception of all frequencies in subsequent months (Park et al., 2010). In comparison with the CaOlaHsd mouse strain, the young adult C57Bl/6 strain expresses significantly different levels of the GluN2A and GluN2B subunits of the NMDAR, metabotropic glutamate receptors and GABA receptors in cortex and hippocampus (Beckmann et al., 2020). All of these are plasticity-related neurotransmitter receptors that are known to contribute to the induction and/or early maintenance of hippocampal LTP, as well as to hippocampus-dependent learning and memory (Izquierdo and Medina, 1995; Mukherjee and Manahan-Vaughan, 2013; Nicoll, 2017). In this context, it is perhaps not that surprising that C57Bl/6 mice also exhibits deficits in item-place memory compared to age-matched CaOlaHsd mice (Beckmann et al., 2020). It bears mentioning, however, that this kind of spatial learning is dependent upon activation of D1/D5R (Lemon and Manahan-Vaughan, 2006).

### Final remarks

In conclusion, we show here that the magnitude of LTP in the hippocampus of freely behaving mice differs greatly depending on the strain. Furthermore, although D1/D5Rs are involved in both LTP and STP in both strains, the effects of pharmacological receptor modulation are more potent in the strain with the larger plasticity responses, namely the CaOlaHsd mouse strain. The different degree of modulation by D1/D5R of hippocampal synaptic potentiation in CaOlaHsd and C57Bl/6 may explain, in part, strain-dependent differences in synaptic plasticity in the murine hippocampus.

## Data availability statement

The raw data supporting the conclusions of this article will be made available by the authors, upon reasonable request.

## Ethics statement

This animal study was reviewed and approved by the Landesamt für Arbeitsschutz, Naturschutz, Umweltschutz und Verbraucherschutz, Northrhine Westphalia, Germany.

## Author contributions

DM-V and MS designed the study. HH and MS conducted the electrophysiological experiments. HH, MS, and DM-V analyzed the data. AL performed the immunohistochemistry and analyzed the data. HH and DM-V wrote the manuscript, including contributions from all authors.

## Footnotes

1
https://github.com/JetiLab/blotit
